# Microenvironment Remodeling Self-Healing Hydrogel for Promoting Flap Survival

**DOI:** 10.34133/bmr.0001

**Published:** 2024-02-22

**Authors:** Yikun Ju, Pu Yang, Xiangjun Liu, Zhihua Qiao, Naisi Shen, Lanjie Lei, Bairong Fang

**Affiliations:** ^1^ Department of Plastic and Aesthetic (Burn) Surgery, The Second Xiangya Hospital, Central South University, Changsha, Hunan 410011, China.; ^2^ Institute of Translational Medicine, Zhejiang Shuren University, Hangzhou, Zhejiang 310015, China.

## Abstract

Random flap grafting is a routine procedure used in plastic and reconstructive surgery to repair and reconstruct large tissue defects. Flap necrosis is primarily caused by ischemia–reperfusion injury and inadequate blood supply to the distal flap. Ischemia–reperfusion injury leads to the production of excessive reactive oxygen species, creating a pathological microenvironment that impairs cellular function and angiogenesis. In this study, we developed a microenvironment remodeling self-healing hydrogel [laminarin–chitosan-based hydrogel-loaded extracellular vesicles and ceria nanozymes (LCH@EVs&CNZs)] to improve the flap microenvironment and synergistically promote flap regeneration and survival. The natural self-healing hydrogel (LCH) was created by the oxidation laminarin and carboxymethylated chitosan via a Schiff base reaction. We loaded this hydrogel with CNZs and EVs. CNZs are a class of nanomaterials with enzymatic activity known for their strong scavenging capacity for reactive oxygen species, thus alleviating oxidative stress. EVs are cell-secreted vesicular structures containing thousands of bioactive substances that can promote cell proliferation, migration, differentiation, and angiogenesis. The constructed LCH@EVs&CNZs demonstrated a robust capacity for scavenging excess reactive oxygen species, thereby conferring cellular protection in oxidative stress environments. Moreover, these constructs notably enhance cell migration and angiogenesis. Our results demonstrate that LCH@EVs&CNZs effectively remodel the pathological skin flap microenvironment and marked improve flap survival. This approach introduces a new therapeutic strategy combining microenvironmental remodeling with EV therapy, which holds promise for promoting flap survival.

## Introduction

Flap grafting is a routine procedure used in reconstructive plastic surgery to repair defective tissues. In patients with large tissue defects, flap grafting remains an irreplaceable treatment modality and involves grafting a flap from a normal region of the patient to the defective area to prevent necrosis [1]. Excessive reactive oxygen species (ROS) generated by ischemia–reperfusion (I/R) leads to oxidative stress, which remains a major cause of flap necrosis [2–4]. In addition, inadequate blood flow to the distal part of the flap is a major cause of flap necrosis. Prevention of I/R injury and establishment of blood supply contribute to flap survival [2–4]. Thus, the development of an effective therapeutic strategy to attenuate or prevent potential I/R injury after flap grafting, while promoting flap blood supply expansion, is important to promote flap survival.

Nanozymes, a rising star in the field of nanomaterials and chemistry in recent years, have been found to have activities similar to those of natural enzymes, thus attracting much attention in the biomedical field [5,6]. Because of their broad range of enzyme-like activities, nanozymes can scavenge overgenerated ROS, thereby remodeling the abnormal oxidative stress microenvironment, and have been shown in studies to treat or prevent a variety of diseases [5,6]. Hou et al. [2] selected Prussian blue nanozymes and applied them topically to flap grafts to verify their role in improving the microenvironment of flap grafts and promoting flap survival. The results showed that Prussian blue nanozymes attenuated oxidative stress and related inflammatory responses, protected vascular endothelial cells, and promoted flap survival.

Ceria nanozymes (CNZs) have multiple enzyme-like activities, including peroxidase (POD), oxidase, catalase (CAT), and superoxide dismutase (SOD), and importantly have low biotoxicity and high biocompatibility [7]. Their different enzyme-like activities depend mainly on their highly reversible Ce^3+^/Ce^4+^ redox ability and surface structure [7]. CNZs have strong ROS-scavenging ability and are expected to play an important role in remodeling the disease microenvironment. Furthermore, research has demonstrated that CNZs possess potential antimicrobial capabilities, attributed to their ability to disrupt the charge profile of bacterial cell surfaces. This characteristic confers CNZs with broad-spectrum antimicrobial properties [8]. However, it is worth noting that oxidative stress is only one of the factors contributing to disease, and nanozymes may be more suited to a supporting role [9,10]. Therefore, combining nanozymes with other bioactive substances or drugs may result in synergistic therapeutic effects.

Extracellular vesicles (EVs) are vesicle-like structures released by cells encasing thousands of proteins, mRNAs, microRNAs, and long noncoding RNAs [11–13]. Mesenchymal-stem-cell-derived EVs are currently a hot research topic in the field of regenerative medicine and play a strong role in promoting tissue repair and regeneration [12,14]. They act directly or indirectly on target cells to activate relevant signaling pathways, exerting tissue repair and regenerative effects [15]. It is well documented that EVs have potent biological activities to promote tissue repair and regeneration, and loading of EVs with hydrogels enables sustained release of EVs at the tissue localization, thus exerting stronger biological activities [16]. Some studies have shown that EVs can promote flap survival mainly by promoting angiogenesis and reducing I/R injury. However, the existing literature is based on the direct application of EVs, and there are no reports on using EV-loaded hydrogel to promote flap survival.

Hydrogels are an excellent drug carrier and extremely biocompatible, allowing CNZs and EVs to be loaded together [17–20]. Self-healing hydrogels, characterized by their injectability and intrinsic self-cross-linking properties upon fracture, primarily operate through reversible dynamic covalent bonding or noncovalent interactions [18,21]. These hydrogels exhibit substantial potential for drug delivery and are widely used in tissue regeneration. They possess the unique ability to self-heal and adapt to defective tissues and organs, providing a protective effect [21]. Under high-shear stress conditions, these hydrogels temporarily transition to a fluid state, reverting back to their original gel form upon the cessation of stress [21], facilitating their administration via syringe injection. Diels–Alder, Schiff base, and thiol–disulfide exchange reactions have been widely researched as major principles in the preparation of self-healing hydrogels. Chitosan, derived from the shells of crustaceans, is a common polysaccharide material with good biocompatibility and antibacterial ability and has been extensively researched in the biomedical field, especially in the application of trauma healing [22]. Laminarin (LAM) is an abundant, nontoxic, degradable polysaccharide found in marine organisms with good biocompatibility and certain anti-inflammatory, antioxidant, and antitumor abilities [23]. Zargarzadeh et al. [24] constructed a photocrosslinked hydrogel based on LAM for cell culture, and the results showed that the degradation products of LAM can provide necessary nutrients to the cells, which, in turn, enhances their viability. LAM is a natural polysaccharide with important research prospects in the biomedical field, but studies on LAM-based hydrogel systems are still scarce.

We propose to utilize the excellent drug-carrying capacity of hydrogels to integrate CNZs and mesenchymal-stem-cell-derived EVs to construct a self-healing hydrogel that can remodel the cellular microenvironment and has a strong ability to promote tissue repair, and validate its function with in vitro and in vivo experiments.

## Materials and Methods

### Experimental design

**Fig. 1.**
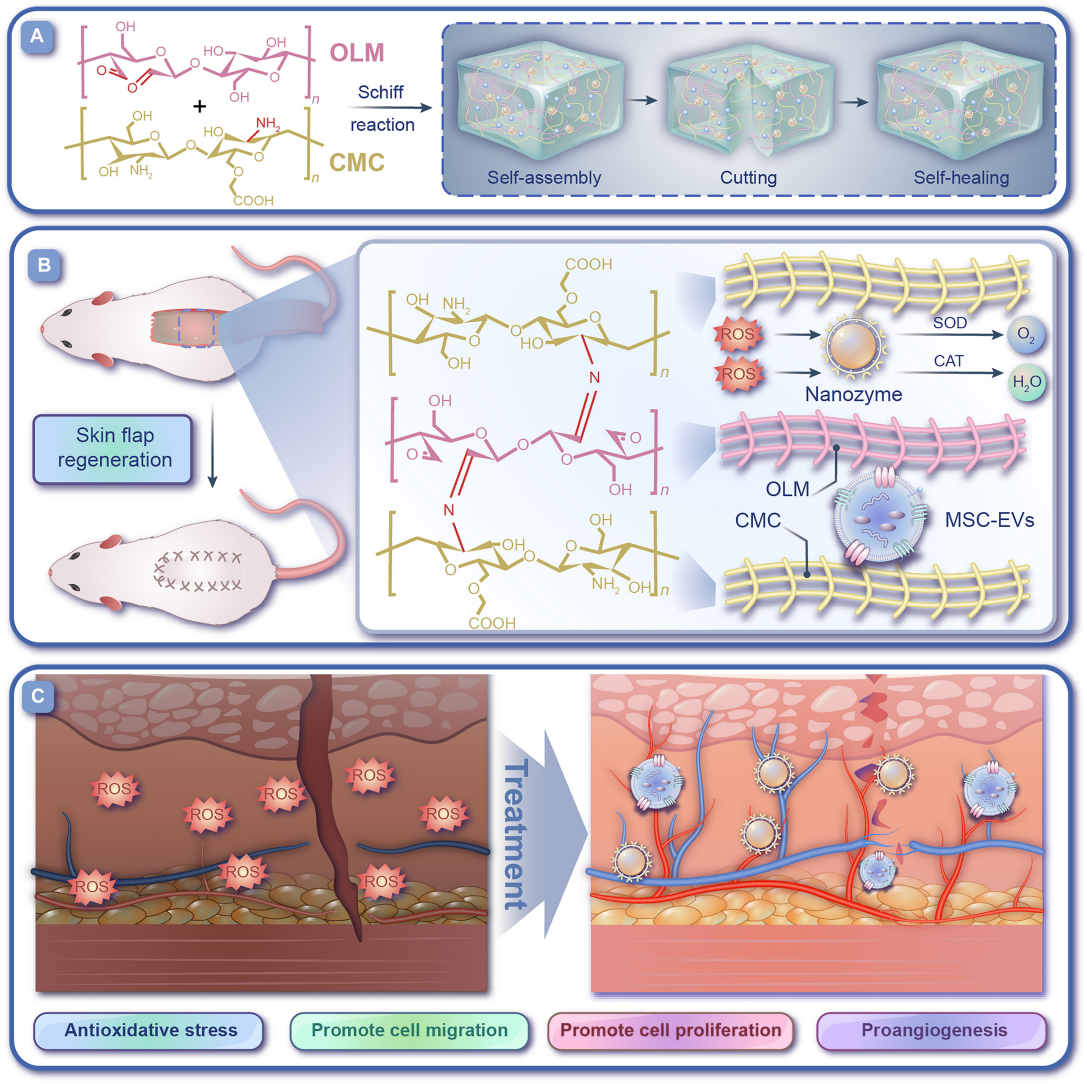
Schematic illustrations of LCH@CNZs&EVs to remodel the microenvironment and promote skin flap survival. (A) The construction of self-healing hydrogel (LCH). (B) Mechanism of action of LCH@CNZs&EVs. (C) Schematic illustration of the remodeling of the flap microenvironment and skin flap healing.

In this study, we constructed a natural self-healing hydrogel [LAM–chitosan-based hydrogel (LCH)] based on the Schiff base reaction by oxidizing LAM (OLM) and carboxymethyl chitosan (CMC). Chitosan is a rare natural polysaccharide that carries an amine group and thus has a unique advantage [25,26]. As chitosan carries its own amino group, it can be used as a good substrate for the Schiff base reaction. However, chitosan is relatively difficult to solubilize in physiological pH environments; hence, we chose CMC. The LCH was loaded with CNZs and mesenchymal-stem-cell-derived EVs (LCH@CNZs&EVs) to promote skin flap healing (Fig. 1). The main function of CNZs is to scavenge excess ROS and improve the pathological microenvironment of skin flap tissues. By contrast, EVs exert a variety of biological activities, such as promoting cell migration, proliferation, and angiogenesis. Improvement in the local microenvironment was accompanied by a synergistic effect that promoted tissue regeneration. To date, no publications show a similar therapeutic strategy for flap grafting; therefore, this study provides a novel therapeutic strategy with great research potential.

### Materials

LAM (molar mass, 470.42), CSC (carboxymethyl chitosan; molar mass, 543.52), NaIO_4_, and ethylene glycol were purchased from Mackin (Shanghai, China). Cerium oxide nanoparticles were purchased from XFNANO (Nanjing, China). Matrigel was purchased from Xiamen Mogengel (Xiamen, China). ROS assay, 3,3′,5,5′-tetramethylbenzidine (TMB), and SOD assay kits were purchased from Beyotime Biotechnology (Shanghai, China). Calcein-AM (calcein acetoxymethyl ester)/propidium iodide (PI) and Cell Counting Kit-8 (CCK8) were purchased from Applygen (Beijing, China). Mesenchymal stem cells (MSCs), human skin fibroblasts (HSFs), and human umbilical vein endothelial cells (HUVECs) were obtained from Procell Life Science & Technology Co. (Wuhan, China). Dulbecco’s modified Eagle medium (DMEM), phosphate-buffered saline (PBS) buffer, fetal bovine serum (FBS), and 0.25% trypsin were obtained from Thermo Fisher Scientific (Waltham, MA, USA). Hematoxylin and eosin (HE) staining kits were obtained from Servicebio (Wuhan, China). All antibodies were purchased from Affinity Biosciences (Jiangsu, China). Male Sprague–Dawley (SD) rats (300 to 350 g) were purchased from Slaughter Kingda Laboratory Animal Co. (Hunan, China).

### Preparation of OLM

LAM (1 g) was dissolved in deionized water (100 ml). Then, NaIO_4_ aqueous solution (10%, w/v) was added and stirred for 24 h in the dark. The reaction was stopped by the addition of ethylene glycol (5 ml). The product was dialyzed in a dialysis bag (3,000 Da) with deionized water for 72 h and then recovered by freeze-drying to obtain OLM.

### Hydrogel preparation and characteristics

OLM and CMC solutions were prepared in PBS buffer. Equal volumes of OLM solutions at concentrations of 6%, 12%, and 24% and CMC solutions at 3%, 6%, and 12% were combined, initiating a Schiff base reaction to form hydrogels. The resultant hydrogels, denoted as LCH-1 (6% OLM:6% CMC), LCH-2 (12%:6%), LCH-3 (24%:6%), LCH-4 (24%:3%), and LCH-5 (24%:12%), were modified by adding various components such as CNZs and EVs to the PBS. The OLM was dissolved in this modified PBS and then mixed with the CMC solution to generate hydrogels with diverse compositions.

#### Self-healing test

Two capsule-shaped LCH hydrogels were prepared and doped with yellow and blue dyes to verify their self-healing abilities. Each hydrogel was cut into two-halves, and one-half containing the yellow dye was closely aligned with the blue-dye counterpart. The self-healing of the hydrogels was evaluated after 1 h of placement at 37 °C.

The hydrogel was subjected to oscillatory strain alterations ranging from 1% to 200% over 1-min intervals, repeated across 3 transformation cycles. At 200% strain, a decrease in *G*′ below the *G*″ indicated hydrogel fracture. However, *G*′ immediately returned to its original value once the strain was reduced back to 1%.

#### Shear-thinning analysis

The change in the viscosity of the hydrogel at shear rates varying from 0.0997 to 100 (1/s) was monitored. A decrease in viscosity with an increasing shear rate or stress characterizes shear-thinning behavior.

#### Mechanical property assessment

Cylindrical hydrogels with a diameter of 9 mm and a height of 3 mm were prepared. Their mechanical properties were measured using a universal testing machine under predetermined parameters.

#### In vitro degradation study

The formulated hydrogel was placed in a glass vial with 1 ml of PBS and incubated at 37 °C. At designated time intervals, the liquid in the bottle was removed, and the weight was recorded.

### Isolation and characterization of EVs

MSCs of generations 3 to 8 were selected for the isolation of EVs. MSCs were cultured in DMEM with 10% FBS, and cells were passaged at 80% to 90% confluency. MSCs were starved for 48 h using serum-free DMEM, and cell culture supernatants were harvested for EV isolation. First, the cell culture supernatant was centrifuged at 300*g* for 15 min to remove dead cells and large cell debris. After removal of the precipitate, the supernatant was centrifuged for 15 min at 3,000*g* to remove smaller cellular debris. The supernatant was carefully removed and centrifuged at 100,000*g* for 1.5 h using an ultracentrifuge; the resulting precipitate contained the EVs.

After determining the concentration of EVs using the BCA (bicinchoninic acid assay) protein quantification kit, they were stored at −80 °C until used for subsequent experiments. The EVs were analyzed for particle size using nanoparticle tracking analysis. After negative staining of EVs, their morphology was observed using transmission electron microscopy (TEM). We verified the markers of EVs using Western blotting (WB). Appropriate markers were selected in accordance with MISEV (minimal information for studies of EVs): 2 positive markers (CD9 and CD81) and 1 negative marker (calnexin).

### Western blotting

Protein quantification of EVs or cells was performed using a BCA protein quantification kit. Next, 30 μg of protein was loaded onto a 4% to 20% FuturePAGE gel (ACE Biotechnology, Nanjing, China), and after electrophoresis, the proteins were transferred onto polyvinylidene fluoride membranes. The membranes were blocked using 5% nonfat milk at 26 °C for 1 h before overnight incubation with the primary antibodies (1:1,000; CD9, CD81, and calnexin) at 4 °C. The membranes were washed thrice with tris-buffered saline containing 0.1% Tween 20 before incubating for 1 h with a secondary antibody (1:10,000; horseradish-peroxidase-linked anti-rabbit) at 37 °C. After washing thrice with tris-buffered saline containing 0.1% Tween 20, images were acquired and analyzed using the Vilber FUSION FX6 Edge imaging system (Eberhardzell, Germany).

### Enzyme-like activity of CNZs

The POD-like activity of CNZs was investigated using the TMB oxidation assay, according to the manufacturer’s instructions. The absorbance values were measured at 650 nm after the reaction of different concentrations of CNZs with TMB at 37 °C for 1 h. A total of 1 mM H_2_O_2_ and different concentrations of CNZs were mixed in 5 ml of deionized water, and O_2_ generation was observed directly. The SOD-like activity of CNZs was measured using an SOD assay kit, according to the manufacturer’s instructions. The absorbance values were measured at 450 nm after different concentrations of CNZs were incubated with the SOD reagent working solution at 37 °C for 30 min.

### Intracellular ROS-scavenging ability

HUVECs were used to quantify the intracellular ROS-scavenging ability. The cells were incubated with hydrogel leachate medium for 4 h, then washed with PBS, and incubated with DCFH-DA (2′,7′-dichlorodihydrofluorescein diacetate): DMEM (1:1,000) for 15 min in the dark. The cells were then washed again with PBS to remove excess DCFH-DA. Cells were treated with 1 mM H_2_O_2_ for 10 min. After washing with PBS again, ROS levels in the cells were captured with a fluorescence microscope and monitored with a microplate reader. DCFH-DA will be excited to generate green fluorescence in the presence of ROS.

### Cell cytotoxicity

The prepared LCHs were soaked in serum-free DMEM at 37 °C for 72 h, and the leachate was then obtained by filtration using a 0.22-μm filter. HUVECs and HSF cells were trypsinized and inoculated in 96-well plates at a density of 10,000 cells per well. The leachate was added to 96-well plates containing the HUVECs or HSF cells and incubated for 48 h. Cell viability was evaluated using calcein-AM/PI staining and the CCK8 assay. The calcein-AM/PI stain was prepared and used according to the manufacturer’s instructions, and images were obtained using a fluorescence microscope. The absorbance values of CCK8 were detected at 450 nm using a microplate reader.

### Cell scratch migration assay

HUVECs and HSF were resuspended in 10% FBS + DMEM medium and inoculated into 12-well plates at 1 ml per well with 200,000 cells. After 24 h of incubation at 37 °C, the cell density was over 90%. The cells were washed twice with PBS, and a straight line was drawn on the culture plate using a 10-μl tip. The cells were then washed twice with prewarmed PBS to remove floating cells. The micrographs were captured after ensuring that the field of view was clean. Solutions with different fractions were added to the cell culture plates, an equal volume of serum-free DMEM was added to the control group, and the micrographs were retaken after 24 h of incubation at 37 °C. After 24 h, images were captured using a microscope. The migration ability of the cells was assessed using ImageJ software [27].

### Tube formation assay

All the materials needed for this experiment should be precooled at 4 °C. Matrigel matrix was spread on a 96-well plate at 50 μl per well and placed at 37 °C for 2 h. Approximately 20,000 HUVECs were then added to each well of the 96-well plate. After culturing the cells for 4 to 6 h, the tubules were stained using a calcein-AM staining solution, and images were captured using a fluorescent microscope. Tubule length was evaluated using ImageJ software.

### Animal experiment

Male SD rats (300 to 350 g) were used to construct the skin flap model. The animal experiments complied with the regulations and were reviewed and approved by the ethical committee of the Second Xiangya Hospital (approval no. 20220637). The rats were anesthetized with isoflurane, and the hair on their backs was removed. A 1.5 cm × 5 cm-sized area was marked on the back of the rat, and the flap was freed using surgical scissors. A thin layer of hydrogel (200 μl) with different compositions was applied to the lower layer of the flap in each experimental group, and an equal amount of saline solution was added to the control group. The flap was then sutured in situ. General imaging and infrared thermography were performed on days 0, 3, 6, and 9. The rats were sacrificed on day 9, and the distal skin flap tissue was analyzed using HE, immunohistochemical, and immunofluorescence staining.

### Biocompatibility in vivo

The prepared composite hydrogel was implanted subcutaneously in SD rats. After 9 days, the rats were sacrificed, and the heart, liver, spleen, lungs, and kidneys were collected to assess their potential organ toxicity. The control group comprised healthy SD rats without any treatment.

### Hemolysis test

The prepared hydrogels were placed in a PBS solution and soaked at 37 °C for 24 h to obtain the soaking solution. Whole blood was obtained after sacrificing the rats and was collected in heparin-containing blood collection tubes. The blood cells were washed several times with PBS to remove damaged cells. The positive control sample was prepared by adding 50 μl of blood cells to 950 μl of distilled water. The negative control was prepared by adding 500 μl of 10% blood cells to an equal volume of PBS. The soaking solutions prepared from the hydrogels were added to the experimental groups. The samples were then incubated at 37 °C for 2 h before each sample was evaluated to determine whether hemolysis had occurred.

### Statistical and graphical analysis

Statistical comparisons between 2 groups were analyzed using the Student’s *t* test, while differences among multiple groups were analyzed through the analysis of variance. All graphs in this study were drawn using OriginPro 2021 software (OriginLab, Northampton, MA, USA). A *P* value of <0.05 was considered statistically significant. Different letters in the chart represent statistically significant differences, whereas identical letters indicate no statistical difference.

## Results

### LCH hydrogel preparation and characterization

**Fig. 2.**
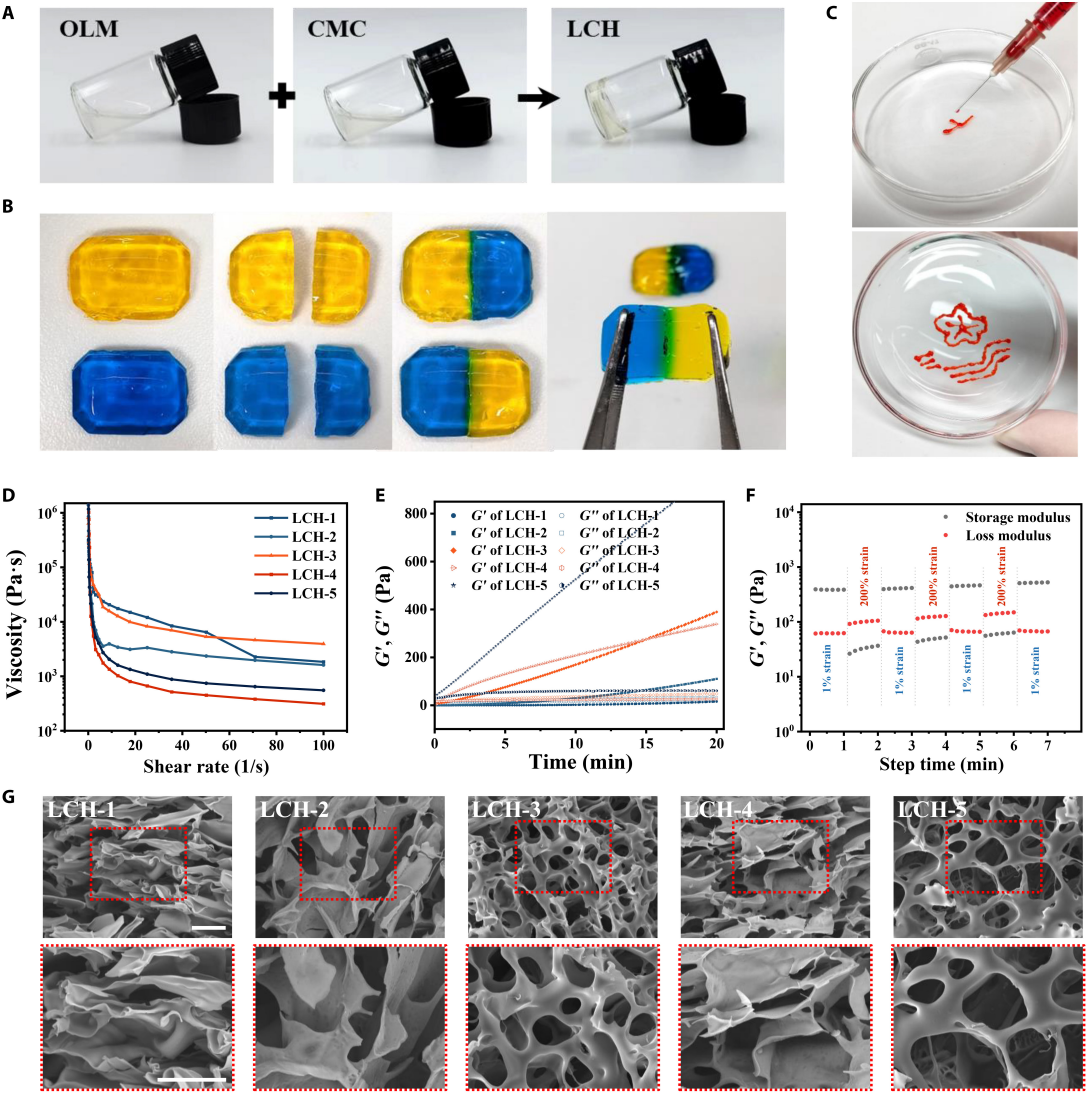
Preparation and characterization of LCHs. (A) OLM and CMC were mixed to form LCH hydrogels. (B) Macroscopic characterization of LCH self-healing properties. (C) Injectability of LCH. (D) The relationship between viscosity and shear rate. (E) Dynamic rheological studies. (F) Step-strain tests of LCH-3 using alternating strain amplitudes of 1% and 200%. (G) SEM images of LCHs. (OLM%:CMC%) LCH-1 = 6:6, LCH-2 = 12:6, LCH-3 = 24:6, LCH-4 = 24:3, and LCH-5 = 24:12. Scale bars, 50 μm.

The LCH hydrogels were prepared on the basis of the principle of the Schiff base reaction. OLM was prepared by the oxidation of LAM using NaIO_4_. The only difference between OLM and LAM is that OLM has a carbonyl group. We examined both LAM and OLM using Fourier transform infrared spectra, in which OLM showed a weak band at 1,730 cm^−1^, supporting the presence of a carbonyl group (Fig. S1). This result was consistent with previous findings reported in the literature [23]. Fourier transform infrared analysis confirmed the successful synthesis of OLM. Subsequent quantification of the oxidation level of OLM, conducted through hydroxylamine hydrochloride titration, indicated an oxidation degree of approximately 36.82 ± 2.97%. The aldehyde (–CHO) group of OLM and the amine group (–NH_2_) of CMC form an imine via a Schiff base reaction, described as a Schiff base bond. Schiff base bonds are reversible and can be broken and formed spontaneously, thus conferring self-healing characteristics to the hydrogels. After mixing equal volumes of OLM and CMC, the formation of a gel rather than a liquid was evident (Fig. 2A). Two capsule-shaped LCH hydrogels were prepared and doped with yellow and blue dyes to verify their self-healing ability. The hydrogels were cut into two-halves, and a part of the hydrogel containing yellow dye was brought into close contact with the blue-dye-containing hydrogel. After 1 h of contact, the 2 parts had healed into a single unit that could withstand a lateral pulling force without breaking (Fig. 2B). We then injected LCH hydrogel through a 1-ml syringe and found that it could easily pass through a 25-gauge needle without clogging (Fig. 2C).

To explore suitable LCH formulations, we prepared 3 different concentrations of LCH. LCH-1, LCH-2, and LCH-3 hydrogels were prepared using 6%, 12%, and 24% OLM solutions, respectively, and equal volumes of 6% CMC solution. We then prepared LCH-4 and LCH-5 hydrogels using 3% and 12% CMC solutions, respectively, each mixed with an equal volume of 24% OLM solution. The injectability of hydrogels is an important prerequisite for their application. Therefore, we evaluated the shear-thinning properties of each LCH group. The hydrogel viscosity decreased with an increasing shear rate at a constant strain of 1%, indicating good shear-thinning properties (Fig. 2D). Gelation time is also critical in hydrogels used for injectable applications; long gel times can cause the solution to spread after injection and before gel formation. Therefore, we measured the gelation times of LCHs. Dynamic rheological studies were also performed for each LCH group, which indicate hydrogel formation when the energy storage modulus (*G*′) is greater than the loss modulus (*G*″). The gelation times for LCH-1, LCH-2, and LCH-3 were 20 min, 8 min, and 35 s, respectively, indicating that the gelation time was closely related to the OLM concentration and that a sufficient number of aldehyde groups were required for the Schiff base reaction (Fig. 2E). An excessively prolonged gelation time can lead to the infiltration of the hydrogel precursor fluid into the tissue interstitial spaces, thereby compromising its efficacy as a drug carrier. Conversely, an overly rapid gelation process may result in premature solidification of the hydrogel at the tip site, thereby hindering procedural execution. In practical applications, a double-barreled syringe is used to concurrently administer OLM aqueous solution and drug-containing CMC aqueous solution. This approach facilitates in situ gel formation directly at the target tissue site. The gel time and better stability of LCH-3 make it more suitable for practical clinical applications. We characterized the mechanical properties of the hydrogel using a universal testing machine, which showed that LCH-3 has relatively good mechanical properties (Fig. S2). In addition, we measured the degradation rate of LCHs in vitro. The results showed that all groups of LCH exhibited commendable degradability. Notably, LCH-1 demonstrated an accelerated degradation rate, which can likely be attributed to its relatively weak cross-linking strength (Fig. S3).

We also determined the rheological self-healing behavior of the LCH-3 hydrogel using continuous step strain measurements. The result indicates that the LCH-3 has good self-healing ability (Fig. 2F). The prepared LCH samples were observed using scanning electron microscopy (SEM). The results show that LCH-3 exhibits a markedly uniform porosity and a complete 3-dimensional mesh structure (Fig. 2G). Considering the practical clinical applications and properties of hydrogels, we chose LCH-3 for the subsequent experiments. Henceforth, the term LCH will specifically refer to LCH-3, unless otherwise specified in the subsequent text.

### Preparation and characterization of LCH@CNZs&EVs

**Fig. 3.**
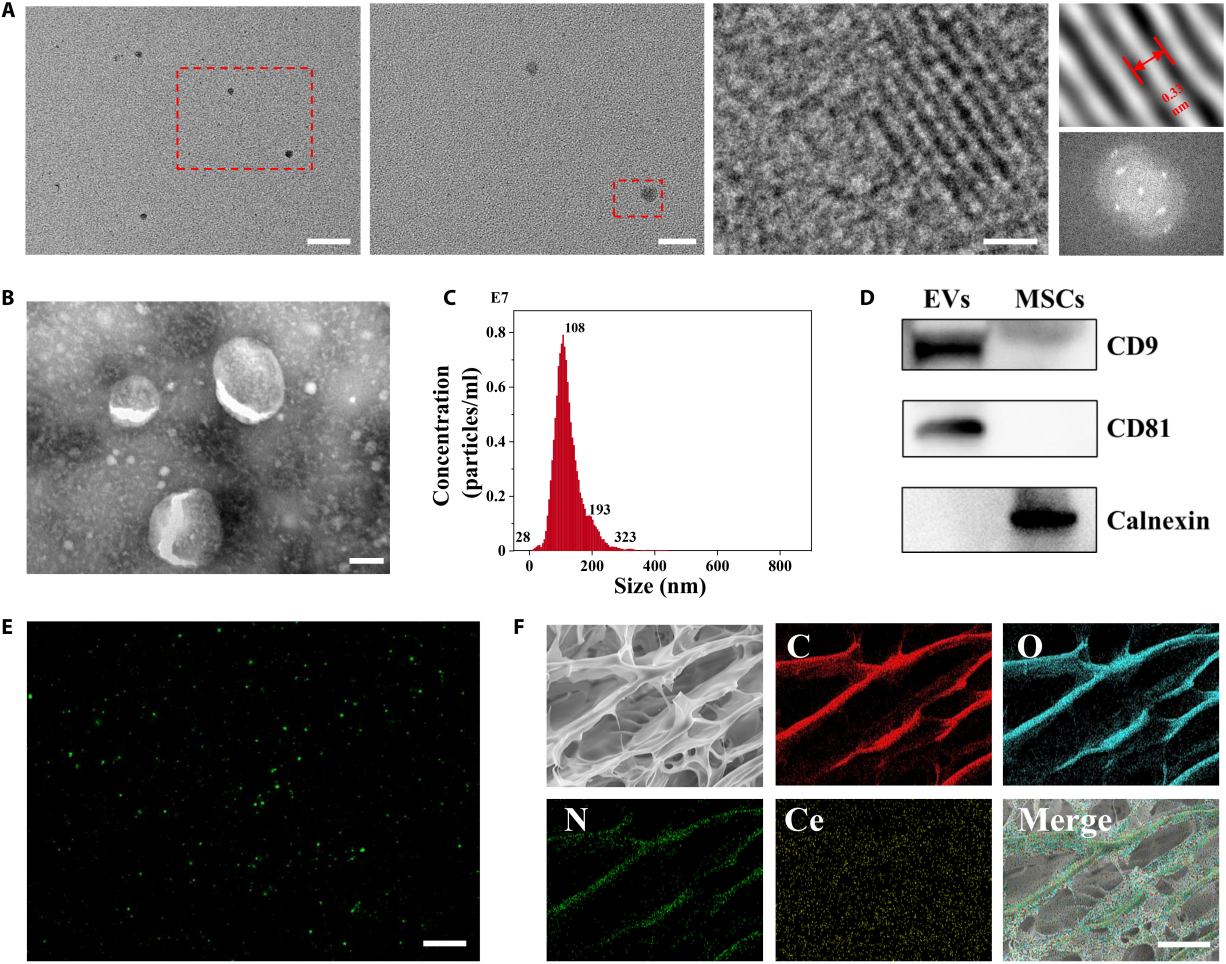
LCH@CNZs&EVs preparation and characterization. (A) TEM images of CNZs. Scale bars, 50, 20, and 1 μm (left to right). (B) TEM images of EVs. (C) Nanoparticle tracking analysis of EVs. Bar scar, 100 μm. (D) WB of EVs. (E) The distribution of PKH67-labeled EVs (green fluorescence) in LCH was observed using confocal laser scanning microscope. Scale bar, 50 μm. (F) SEM and EDS-mapping images of LCH@CNZs&EVs. Scale bar, 50 μm.

The morphology of the CNZs was observed using TEM, and the particle size was relatively homogeneous. Under high magnification, an obvious lattice structure was visible. The selected area electron diffraction pattern of CNZs showed crystallinity (Fig. 3A). Particle size analysis revealed that the particle size of CNZs ranged from 3 to 20 nm and was mainly concentrated around 10.1 nm (Fig. S4). The zeta potential of CNZs was −29.8 mV, confirming their good biocompatibility and absence of any cationic effects (Fig. S5).

EVs were isolated mainly by ultracentrifugation, and TEM observation revealed obvious vesicle-like structures (Fig. 3B), consistent with previous reports [28]. Nanoparticle tracking analysis characterized the isolated EVs, indicating that their particle sizes were concentrated around 108 nm, classifying them as small EVs (Fig. 3C). To confirm the characteristics of EVs, we performed WB analysis, targeting the appropriate markers in accordance with the minimal information for studies on EVs [29], including 2 positive markers (CD9 and CD81) and 1 negative marker (calnexin) (Fig. 3D). The results confirmed the isolation of typical EVs without cellular debris. Subsequently, preliminary experiments were conducted to confirm the biological activity of the prepared EVs. Treatment of HUVECs with different concentrations of EVs exerted significant effects on the cells, including the promotion of cell migration and tube formation in a dose-dependent manner (Figs. S6 and S7), consistent with previous reports on the strong biological activity of EVs [30,31].

We labeled the EVs with PKH67 (a dye for conventional cell membrane labeling) to enable green fluorescence visualization. PKH67, a widely utilized cell membrane stain, is commonly used for EV labeling. We loaded the PKH67-labeled EVs into the LCH and observed them using a confocal laser scanning microscope to demonstrate the distribution of EVs in the LCH (Fig. 3E). After freeze-drying, the microstructure of LCH@CNZs&EVs was evaluated using SEM and EDS (energy dispersive spectrometer)-mapping analyses. EDS-mapping analyses revealed the major elemental compositions present in LCH@CNZs&EVs, demonstrating the presence of cerium (Fig. 3F).

### Antioxidant effect in vitro

**Fig. 4.**
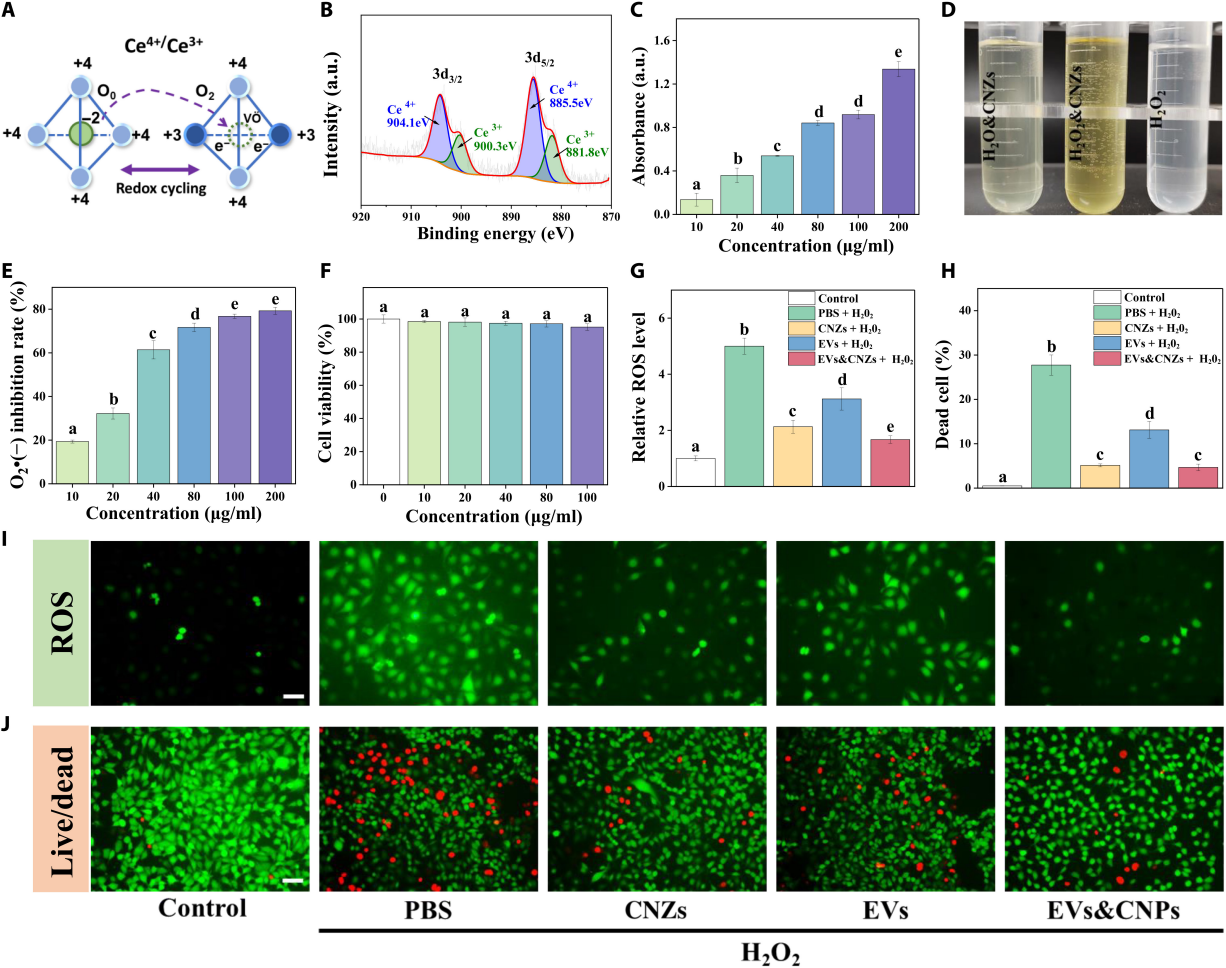
Antioxidant effect in vitro. (A) Illustration of redox cycling of CNZs. (B) X-ray photoelectron characterization. (C) Verification of POD-like activity. (D) Verification of CAT-like activity. (E) Verification of SOD-like activity. (F) Cytotoxicity assay of CNZs. (G and I) Ability of CNZs and EVs to clear ROS. (H and J) Cytoprotective effects of CNZs and EVs in oxidative stress environments. Scale bars, 100 μm. a.u., arbitrary units.

I/R damage inevitably occurs during flap grafting, resulting in excess ROS [32,33]. Therefore, we aimed to verify the enzyme-like ability of CNZs to scavenge excess ROS. After confirming that the conversion of Ce^3+^/Ce^4+^ was the main mechanism of the catalytic reaction in CNZs (Fig. 4A) [7,34], we demonstrated the presence of Ce^3+^ and Ce^4+^ in CNZs by x-ray photoelectron characterization (Fig. 4B). X-ray photoelectron characterization revealed the trivalent and tetravalent states of cerium, which is beneficial to the catalytic reaction of CNZs. Furthermore, we evaluated the enzyme-like activity of CNZs in vitro. POD-like activity was verified using the TMB oxidation assay. As the concentration of CNZs increased, the absorbance value at 650 nm also increased, indicating an increasing concentration effect and demonstrating that CNZs have a POD-like ability (Fig. 4C and Fig. S8).

CAT can catalyze the generation of H_2_O and O_2_ from H_2_O_2_, allowing it to be directly detected using H_2_O_2_. The CAT-like ability of CNZs in the presence of H_2_O_2_ was evaluated, and significant O_2_ bubble generation was observed. By contrast, no bubbles were observed when CNZs were added to airless water or in the H_2_O_2_ negative control. This confirmed that the CNZs had CAT-like activity (Fig. 4D and Fig. S8).

The SOD activity of CNZs was evaluated using the SOD assay kit. As the concentration of CNZs increased, the inhibition of ·O_2_– gradually increased, with an increasing concentration effect, confirming that CNZs have SOD-like activity (Fig. 4E and Fig. S8). ·O_2_– is a strong oxidant that is highly cytotoxic, and the scavenging of ·O_2_– helps reduce the oxidative stress response of cells [35].

To confirm the antioxidant capacity at the cellular level, we evaluated the cytotoxicity of different concentrations of CNZs using HUVECs to determine the appropriate dose. The results of the CCK8 assay showed that even CNZs (100 mg/ml) did not significantly affect cell viability (Fig. 4F). The intracellular ROS-scavenging capacity was assessed using DCFH-DA. Upon cellular uptake, DCFH-DA exhibits green fluorescence in the presence of ROS, thereby enabling the quantification of ROS generation based on the fluorescence intensity. Cells were pretreated for 4 h with different groups before H_2_O_2_ was added for 1 h to induce oxidative stress in the cells. The scavenging efficiency of ROS was observed using fluorescence microscopy. The results showed that both the CNZs and EVs groups scavenged excessive intracellular ROS. However, the CNZs&EVs group showed the strongest ROS-scavenging ability, suggesting a synergistic effect between CNZs and EVs (Fig. 4G and I). Cells were pretreated with different groups for 4 h, after which H_2_O_2_ was added and incubated for 24 h to evaluate the protective effect of each group on the cells under oxidative stress. Cell live/dead staining results showed that all the CNZs, EVs, and CNZs&EVs groups reduced the cell death rate (Fig. 4H and J). CNZs&EVs did not show a significant advantage here, and it is possible that the short term of cell pretreatment or the long term of H_2_O_2_ incubation somewhat attenuated their synergistic effect. Their effect on HUVEC migration under oxidative stress was also evaluated. The results showed that the migration ability of the cells was significantly inhibited under oxidative stress. CNZs&EVs showed a better effect in an oxidative stress environment than that of EVs alone (Fig. S9).

### Promoting cell migration and angiogenesis

**Fig. 5.**
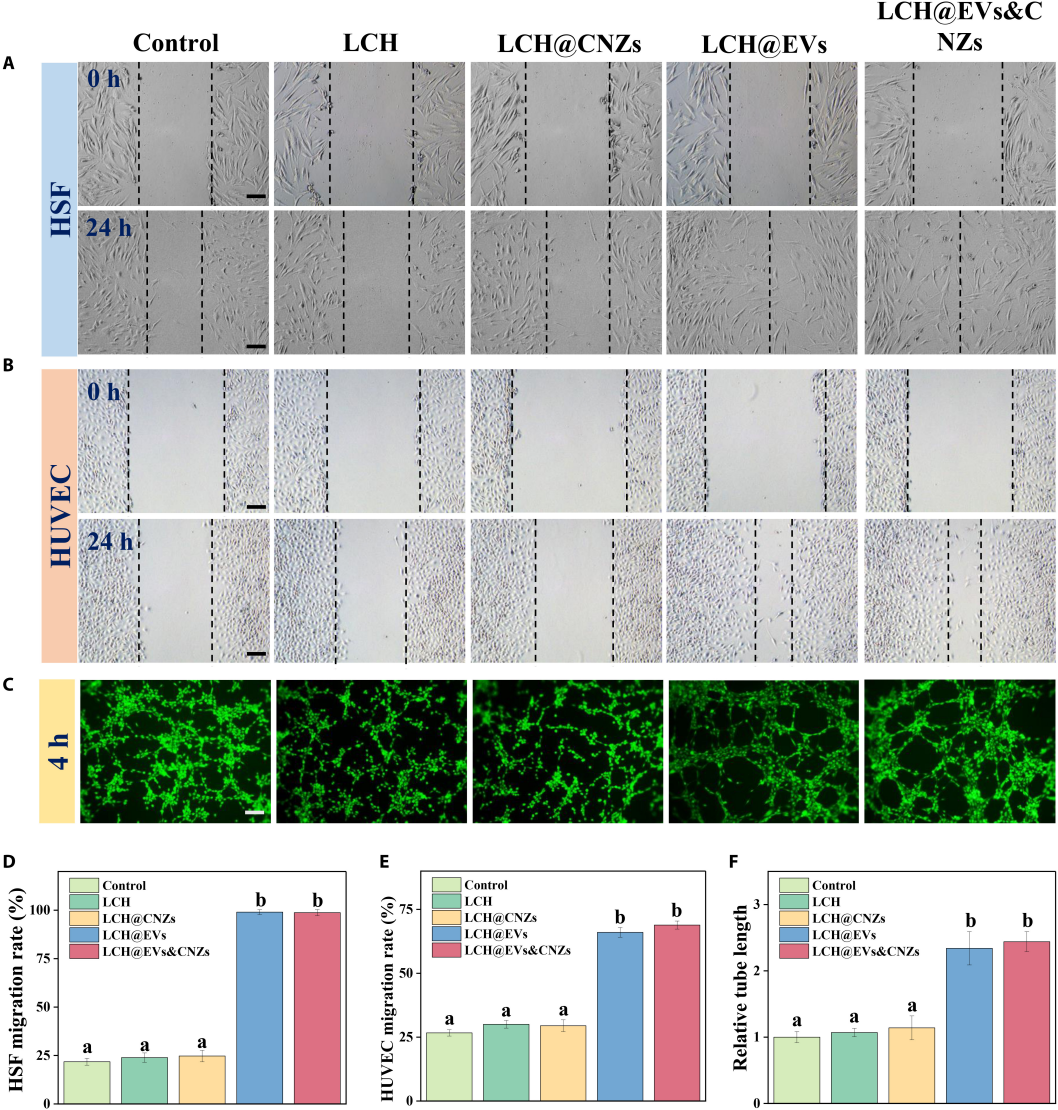
Promoting cell migration and angiogenesis. (A) HSF and (B) HUVEC migration tests. (C) Tube forming ability. (D) HSF migration rate. (E) HUVEC migration rate. (F) Relative tube length. Scale bars, 100 μm.

EVs have important research significance in regenerative medicine and are widely used to promote the repair and regeneration of various tissues and organs [12,36,37]. Therefore, we evaluated the effect of each hydrogel group on cell function using HSFs and HUVECs (Fig. 5A and B). LCH and LCH@CNZs did not promote the migration of HSF and HUVECs; they also showed no inhibitory effect, remaining essentially the same as the control group at baseline. The results of the cell scratch migration assays showed that both LCH@EVs and LCH@CNZs&EVs could significantly promote the migration ability of HSF and HUVECs. There was no statistical difference in the migration-facilitating ability between the LCH@EVs and LCH@CNZs&EVs groups (Fig. 5D and E). The ability of HSF and HUVECs to migrate contributes to the healing of the tissue surrounding the skin flap.

Next, the effect of each group on the tube-forming ability of HUVECs was evaluated (Fig. 5C and F). LCH and LCH@CNZs did not promote tubule formation; however, LCH@EVs and LCH@CNZs&EVs showed the same protubule formation ability. Angiogenesis is crucial for flap survival, and improved blood flow facilitates the removal of cellular metabolites and brings oxygen and nutrients to the distal end of the flap [38,39].

### Promoting the survival rate of skin flaps in vivo

**Fig. 6.**
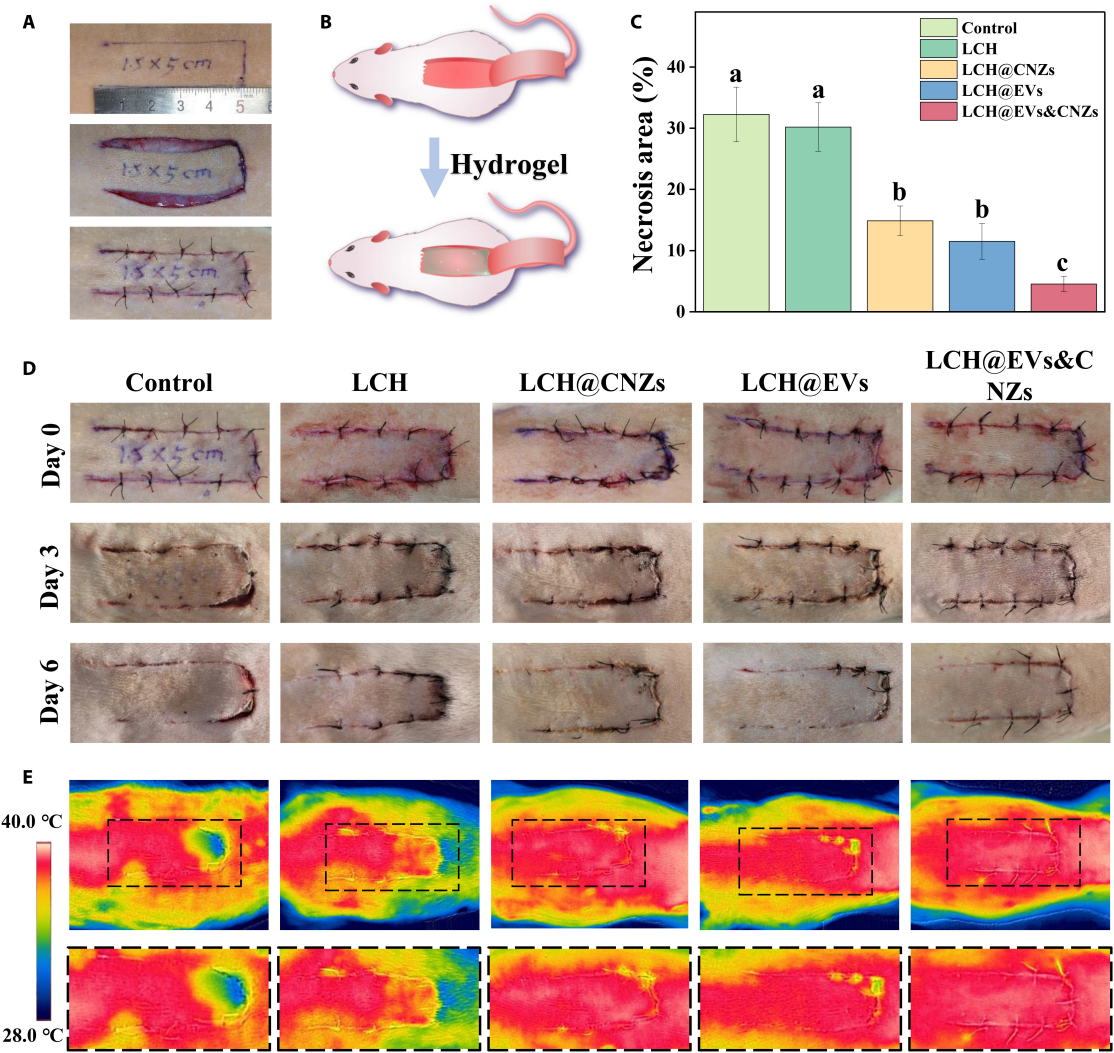
Promoting the survival rate of skin flaps in vivo. (A) Preparation of a randomized skin flap model. (B) Schematic diagram of hydrogel application. (C) Statistics on the relative necrotic area of the skin flap. (D) Representative photos of each group of flaps. (E) Infrared photographs of the skin flap, which can indirectly reflect the skin flap blood flow.

The effect of the hydrogel, LCH@CNZs&EVs, on an SD rat dorsal random pattern flap model was investigated to further demonstrate its ability to promote flap survival in vivo. A 1.5 cm × 5 cm random flap was prepared on the dorsal side of the rat, and the hydrogel was injected into the underside before suturing the flap in situ (Fig. 6A and B). To comprehensively evaluate its survival or necrosis rate, we observed the general condition of the flap was observed and assessed the blood flow using infrared thermography. Both LCH@CNZs and LCH@EVs showed the ability to promote flap survival, indicating that CNZs and EVs play an important role in improving the flap environment and promoting flap survival. However, the relative necrotic area was the smallest in the LCH@CNZs&EVs group (Fig. 6C to E). These results support the efficacy of our hypothesized therapeutic strategy in the rat model, demonstrating that EVs can better promote flap repair after improving the microenvironment using nanozymes.

**Fig. 7.**
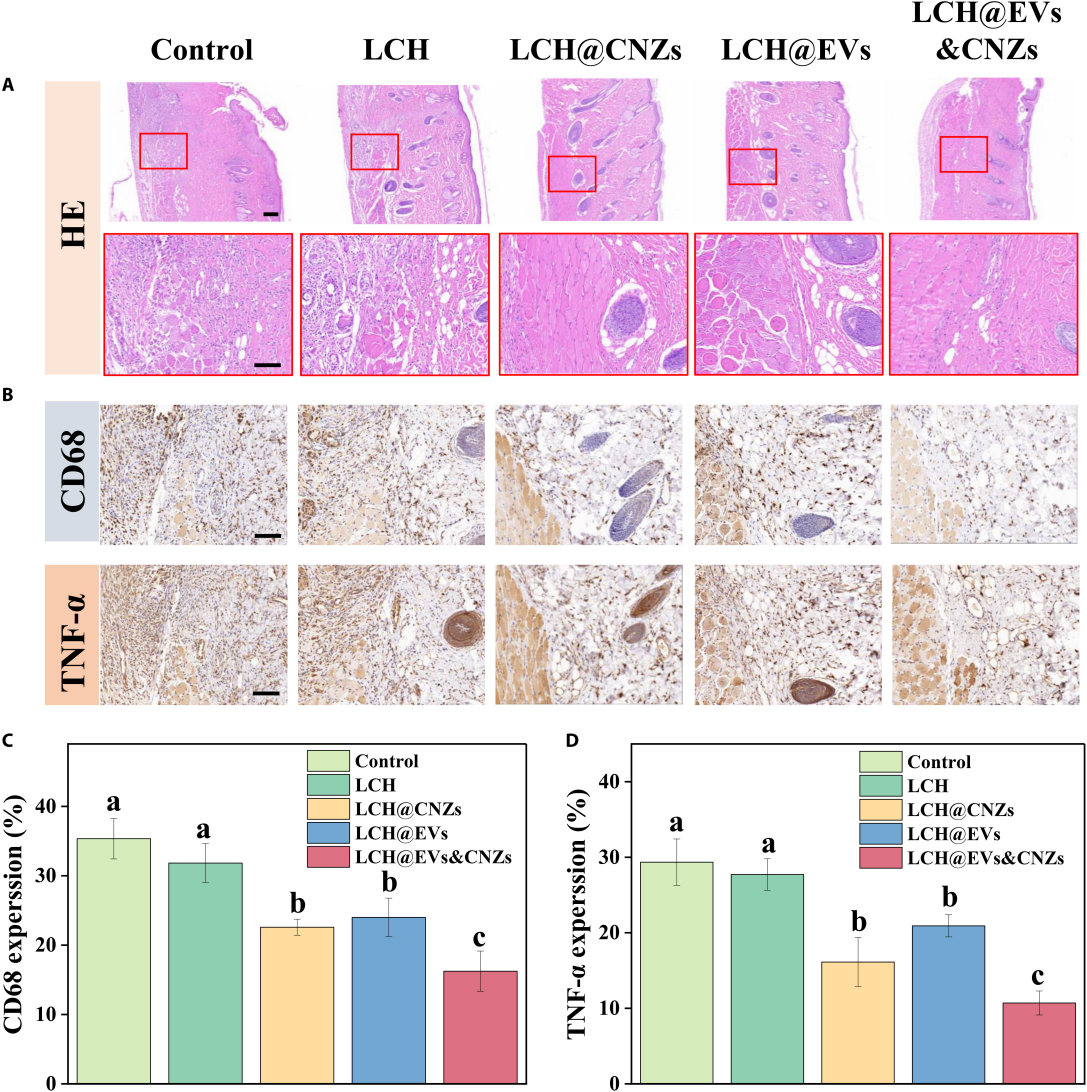
HE staining and immunohistochemical staining. (A) HE staining. (B) Immunohistochemical staining of CD68 and TNF-α. (C) CD68 expression. (D) TNF-α expression. Scale bars, 100 μm.

The distal flap tissue was further evaluated using HE staining (Fig. 7A). Compared with the control and LCH groups, all the LCH@CNZs, LCH@EVs, and LCH@CNZs&EVs groups showed better tissue integrity and clearer structures. To assess the immune microenvironment, we conducted immunohistochemical staining for inflammatory indicators. Previous studies have shown that EVs can improve the immune microenvironment and their role in promoting tissue repair [40]. Nanozymes exert an indirect anti-inflammatory effect, mainly by scavenging excessive ROS [41]. Through histopathological sections, we observed significant infiltration of inflammatory cells and overexpression of the inflammatory factor tumor necrosis factor-α (TNF-α) in the control and LCH groups (Fig. 7B). CD68 is a surface marker for macrophages that flanks the extent of inflammatory cell infiltration [42]. The infiltration of inflammatory cells and the expression of the inflammatory factor TNF-α were decreased in both the LCH@CNZs and LCH@EVs groups, with the anti-inflammatory ability being slightly stronger in the LCH@CNZs group. By contrast, a significant anti-inflammatory effect was observed in the LCH@CNZs&EVs group, suggesting that CNZs and EVs can exert synergistic anti-inflammatory effects in vivo (Fig. 7C and D).

### Promoting the survival rate of skin flaps in vivo

**Fig. 8.**
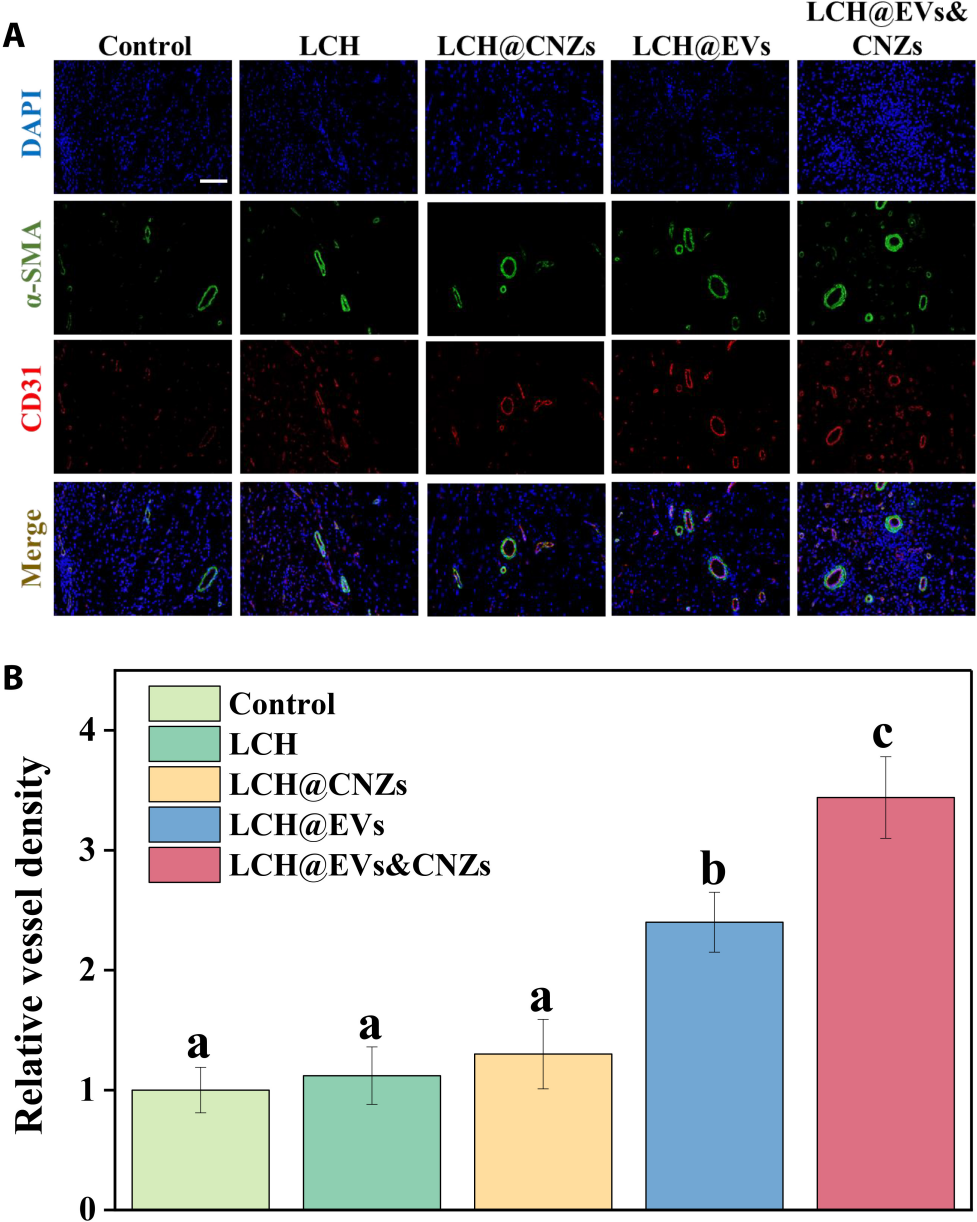
Immunofluorescence staining. (A) Immunofluorescence staining of CD31 and α-SMA. (B) Relative vessel density. Scale bar, 100 μm. DAPI, 4′,6-diamidino-2-phenylindole.

The reconstruction of flap blood flow is essential for flap survival. To assess the role of LCH@CNZs&EVs in promoting angiogenesis in vivo, we determined the level of angiogenesis using immunofluorescence staining for CD31 and α-smooth muscle actin (α-SMA), 2 markers commonly used to assess neovascularization (Fig. 8A). The immunofluorescence results showed a significant increase in the density and extent of neovascularization in the LCH@EVs and LCH@CNZs&EVs groups, with a relatively greater density observed in LCH@CNZs&EVs (Fig. 8B). The angiogenesis effect of EVs has been widely reported and is consistent with our results. Importantly, our results suggest that nanozymes improve the microenvironment and enhance neovascularization by EVs. This clearance of excess ROS, possibly contributed by nanozymes, appears to positively influence neovascularization.

### Biosafety of LCH@CNZs&EVs in vitro and in vivo

The cytotoxicity of LCH@CNZs&EVs was evaluated using live/dead cell staining and the CCK8 assay (Fig. S10). The results showed that neither LCH nor LCH@CNZs&EVs had any significant effect on the survival of both HUVECs and HSF at 48 h. However, the LCH@CNZs&EVs group exhibited promotion of cell proliferation. After the rats were sacrificed, organs were obtained from the control and LCH@CNZs&EVs groups to evaluate potential organ toxicity (Fig. S11). The tissue condition of the heart, liver, spleen, lung, and kidney was evaluated by HE staining. The results showed no tissue or organ abnormalities in the experimental group compared to the tissues in the control group. Furthermore, the hemolysis assay demonstrated that the hydrogels in all groups exhibited good hemocompatibility and did not cause hemolytic reactions (Fig. S12).

## Discussion

Hydrogels have long been the focus of research in the field of biomaterials and play an important role in regenerative medicine. In addition to conventional block hydrogels, self-repairing hydrogels, hydrogel microspheres, and 3-dimensional scaffolds printed on hydrogel-based bioinks have been developed [43–46]. The use of hydrogels to promote flap regeneration has been previously reported in the literature. Zhou et al. [47] constructed a set of programmable integrated and discharged hydrogels with controllable fold patterns to promote the regeneration of randomized flaps, and the hydrogel adhesive could provide sufficient adhesion for tissue sealing and promote neovascularization. Mao et al. [48] developed a self-healing hydrogel for promoting the regeneration of skin flaps by utilizing the principle of Ag-S coordination. A self-healing hydrogel was used to promote flap regeneration with favorable anti-inflammatory, anti-infective, and neovascularization effects. In the present study, we attempted to construct a self-healing hydrogel that could remodel the local oxidative stress microenvironment of the skin flap and, at the same time, have strong biological activity.

Oxidative stress is an imbalance of redox homeostasis in the body due to a variety of factors, which usually manifests itself in the generation of large amounts of ROS, leading to an inflammatory infiltration of neutrophils, an increase in the secretion of proteolytic enzymes, and the production of large amounts of oxidative intermediates [49]. Oxidative stress is a pathological state that leads to reduced or dysfunctional cellular biological functions and is an essential factor in the development of various diseases [49]. Remodeling redox homeostasis is important for disease regression. Currently, there have been a large number of studies confirming that the scavenging of ROS using nanozymes can be used for the prevention or treatment of a wide range of diseases, including inflammatory bowel disease, acute kidney injury, ischemic stroke, diabetic trauma, osteoarthritis, and others. However, one thing that needs to be recognized is that redox homeostasis imbalance plays a relatively limited role in a disease. Relying solely on the removal of ROS is hardly sufficient to achieve a complete treatment of a disease, as it may be only one of the factors influencing disease regression.

It has been demonstrated that EVs, especially those derived from MSCs, have potent bioactivities and can exert effects similar to those of stem cell therapies with lower immunogenicity. EVs are a hot research topic in the field of regenerative medicine and can promote tissue repair and regeneration through various mechanisms [12,14]. Nanozymes can remove excessive ROS induced by I/R injury and improve the cellular microenvironment to enable EVs to play a better role. Therefore, the present study proposes the use of nanozymes as antioxidants to assist EVs in promoting flap regeneration. Nanozymes can scavenge excess ROS caused by I/R injury and improve the cellular microenvironment, allowing EVs to play a better role. A recent important study showed that there is a synergistic effect between nanozymes and EVs, and the combination of the 2 can exert stronger antioxidant and anti-inflammatory effects, which can significantly promote the repair of osteoarthritis [50]. This result suggests that the therapeutic strategy proposed in this study has research and application value in other disease models as well. However, it should be noted that more in-depth mechanistic studies are still lacking, while the potential toxic side effects of nanozymes are also an issue that researchers need to be aware of.

Overall, this study presents a new therapeutic strategy to promote flap survival. We prepared a marine-derived natural polysaccharide self-healing hydrogel using Schiff’s base reaction to load CNZs and EVs to utilize their synergistic effects. Nanozymes scavenge the excess ROS generated in the pathological microenvironment and synergize with EVs to promote tissue regeneration and repair. To date, the biological activity of nanozymes has only been attributed to their excellent antioxidant properties. However, oxidative stress is only one of the influencing factors in the process of tissue repair, and a single antioxidant property cannot satisfy the needs of tissue repair and regeneration. Therefore, combined EVs can exert more biological activities on top of their antioxidant properties, such as promoting cell proliferation, migration, and angiogenesis. In summary, this study proposes a new therapeutic strategy, i.e., the use of hydrogel-loaded nanozymes with EVs, to achieve remodeling of the pathological microenvironment and promote tissue repair, which has a strong research and application prospect in the field of regenerative medicine.

### Ethics Approval and Consent to Participate

The animal experiments complied with the regulations and were reviewed and approved by the ethical committee of the Second Xiangya Hospital (approval no. 20220637).

## Data Availability

The data in this study are available upon reasonable request.
