# Supplementary material for: Microenvironment Remodeling Self-Healing Hydrogel for Promoting Flap Survival
Source: Biomater Res. 2024 Feb 22;28:0001. doi: 10.34133/bmr.0001 (PMC10882600; doi:10.34133/bmr.0001)
Supplement: Supplementary 1 — Figs. S1 to S12 [file bmr.0001.f1.docx]

**Supplementary material**

**Microenvironment** **Remodeling Self-Healing Hydrogel for Promoting Flap Survival**

**Short titles:** Microenvironment Remodeling Hydrogel

Yikun Ju^1^, Pu Yang^1^, Xiangjun Liu^1^, Zhihua Qiao^1^, Naisi Shen^1^, Lanjie Lei^2^*, Bairong Fang^1^*

**Affiliations**

1. Yikun Ju, Pu Yang, Xiangjun Liu, Zhihua Qiao, Naisi Shen, and Bairong Fang*

Department of Plastic and Aesthetic (Burn) Surgery, The Second Xiangya Hospital, Central South University, Changsha, Hunan, 410011, China.

^*^Address correspondence to: [fbrfbr2004@csu.edu.cn](mailto:fbrfbr2004@csu.edu.cn)

2. Lanjie Lei*

Institute of Translational Medicine, Zhejiang Shuren University, Hangzhou, Zhejiang, 310015, China.

^*^Address correspondence to: leilanjie1988@163.com


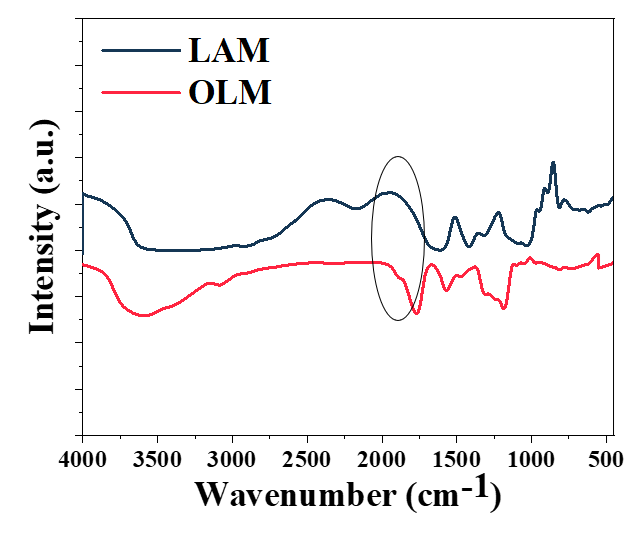


**Fig. S1 Fourier transform infrared (FTIR) spectra of LAM and OLM.**


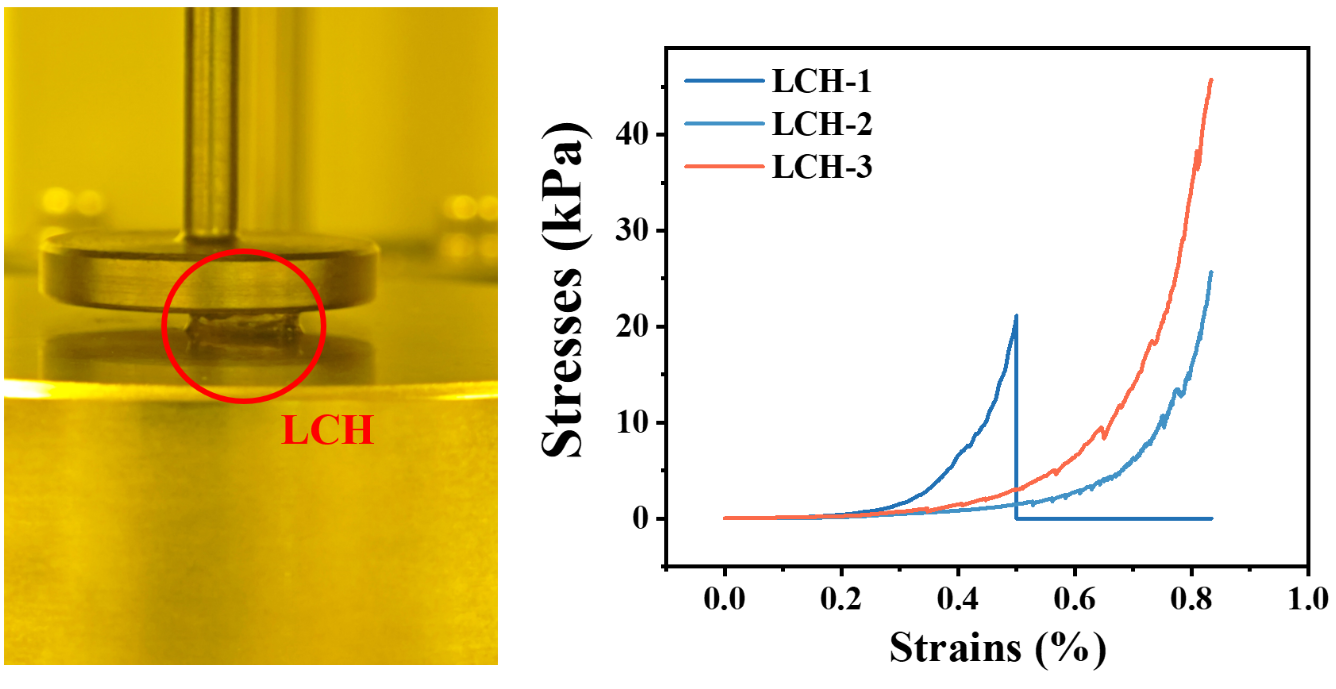


**Fig. S2 The compression modulus of LCHs were measured using a Universal Testing Machine.**


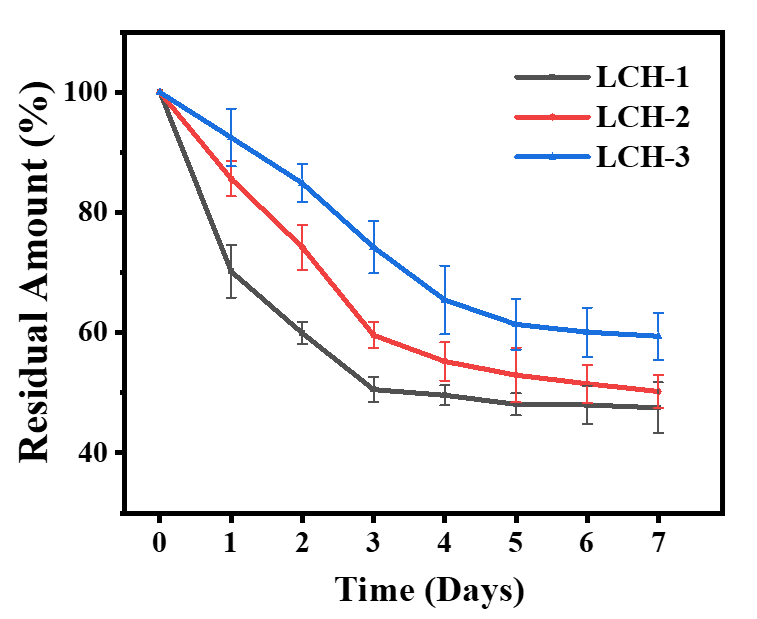


**Fig. S3 *In vitro* degradation curve of LCHs.**


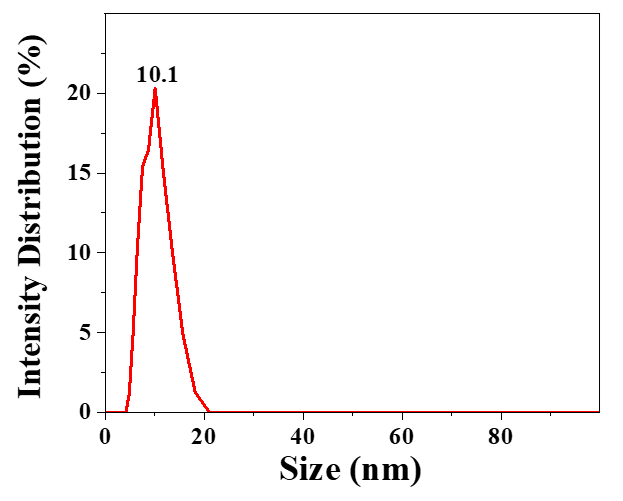


**Fig. S4 Particle size analysis of CNZs.**


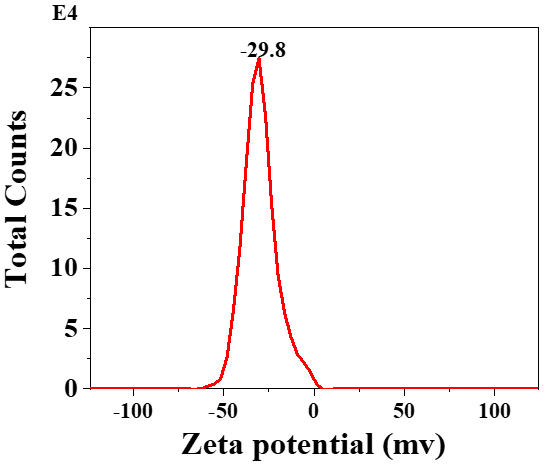


**Fig. S5 The zeta potential of CNZs.**


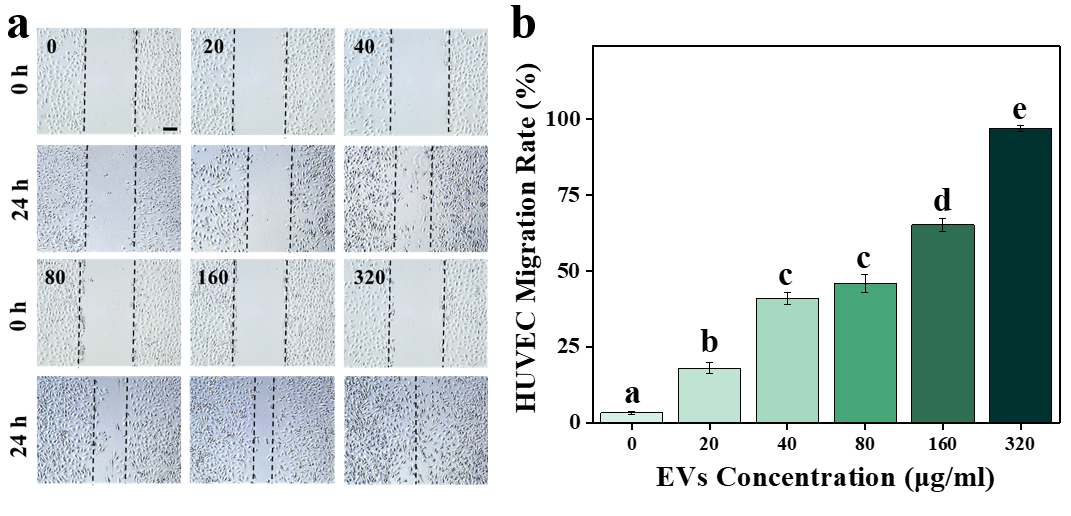


**Fig. S6 (a) HUVEC cell migration assay with different concentrations of EVs. (b) The migration rate of HUVEC. The scale bar is 100 μm.**


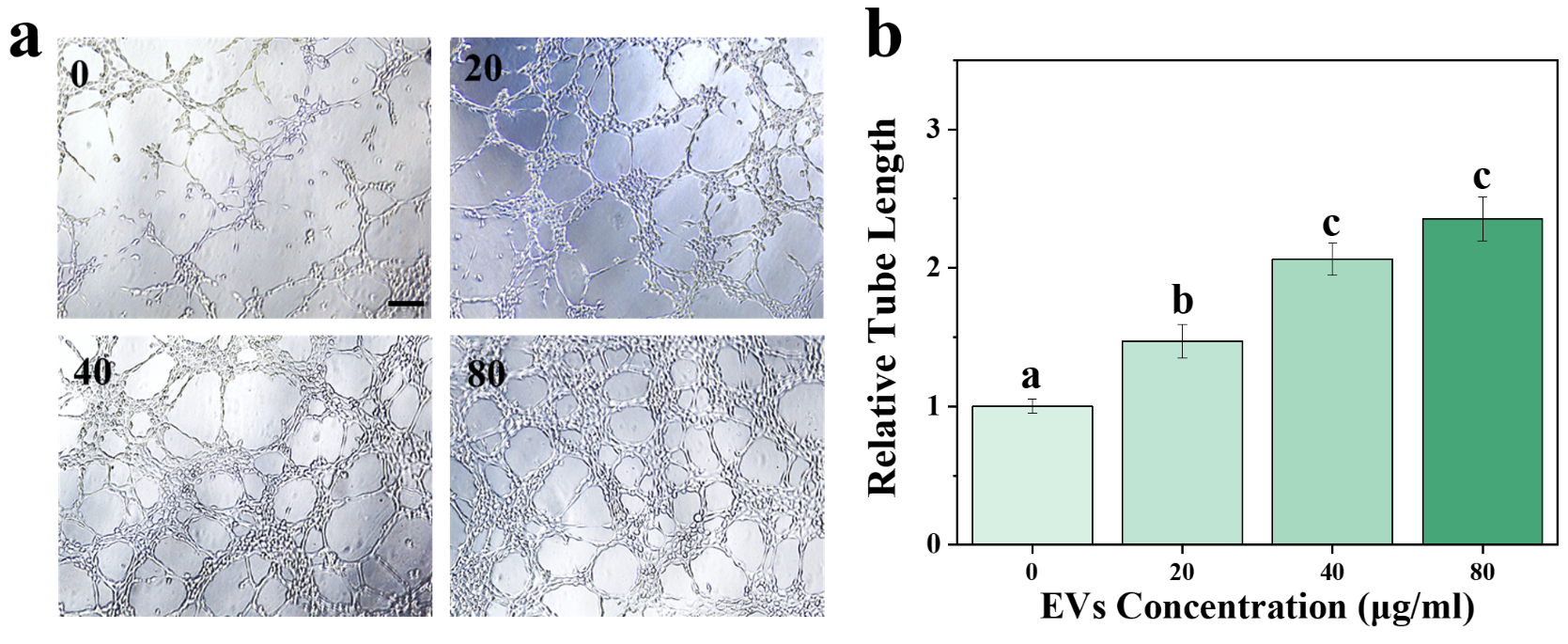


**Fig. S7 (a) HUVEC tube formation assay with different concentrations of EVs. (b) The relative tube length of HUVEC. The scale bar is 100 μm.**


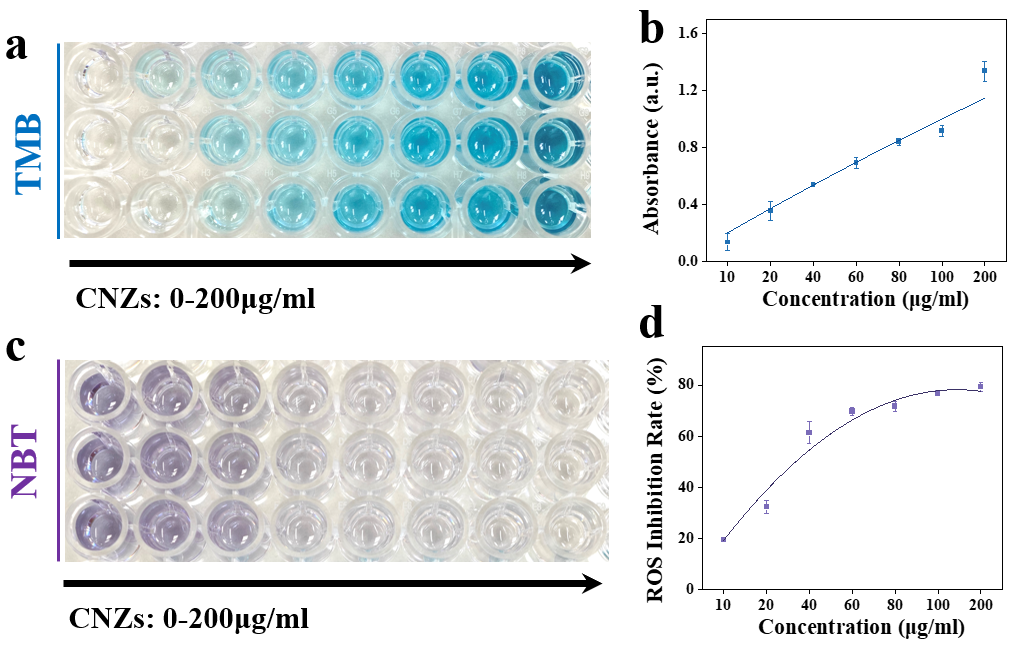


**Fig. S8 (a, b)** **The 3,3′,5,5′-tetramethylbenzidine (TMB) oxidation assay. (c, d) The total superoxide dismutase assay kit with nitro-blue tetrazolium (NBT).**


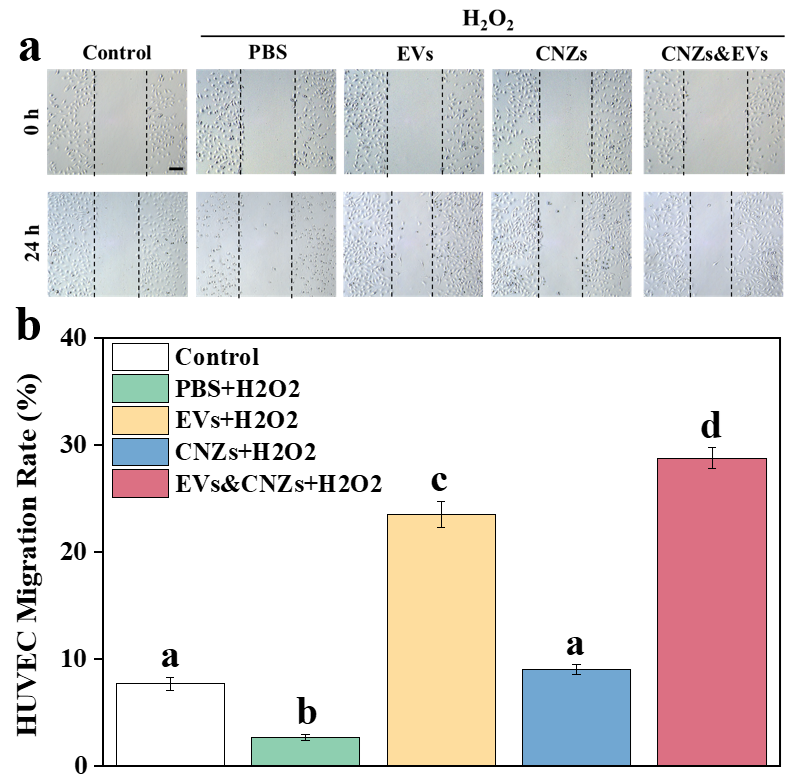


**Fig. S9 (a) HUVEC cell migration assay under H2O2 with different groups. (b) The migration rate of HUVEC. The scale bar is 100 μm.**


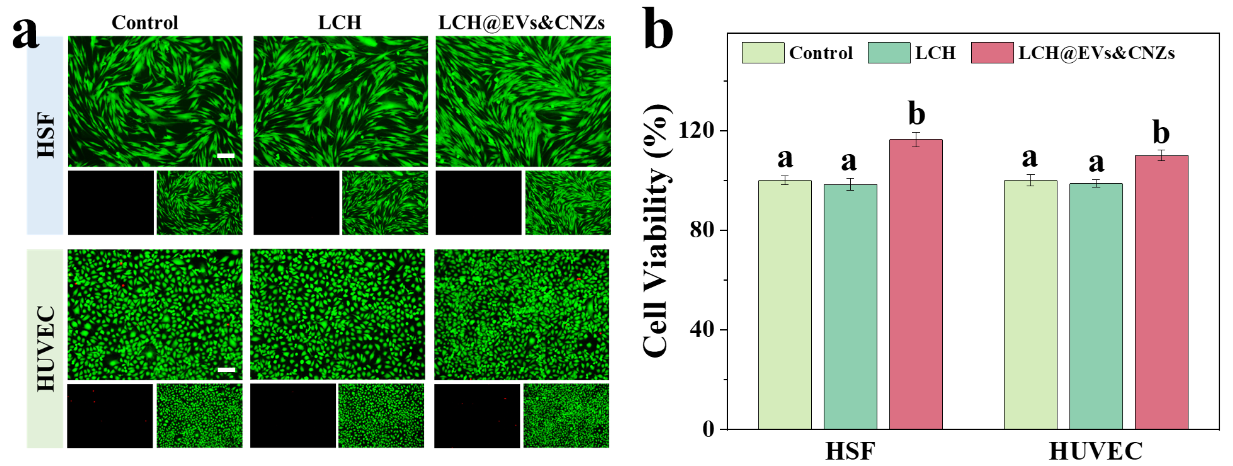


**Fig. S10 (a) HSF and HUVEC cell live/dead staining images at 48h. (b) HSF and HUVEC cell viability at 48h. The scale bar is 100 μm.**


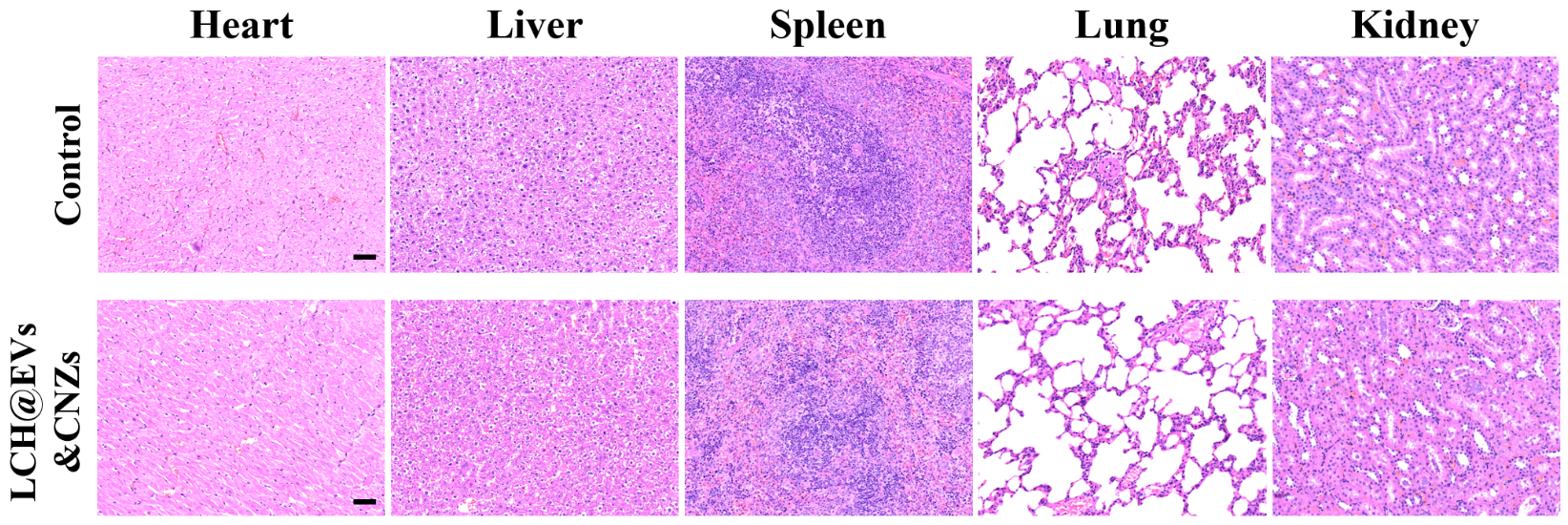


**Fig. S11 Organ toxicity test.** HE staining of heart, liver, spleen, lung and kidney. The scale bar is 50 μm.


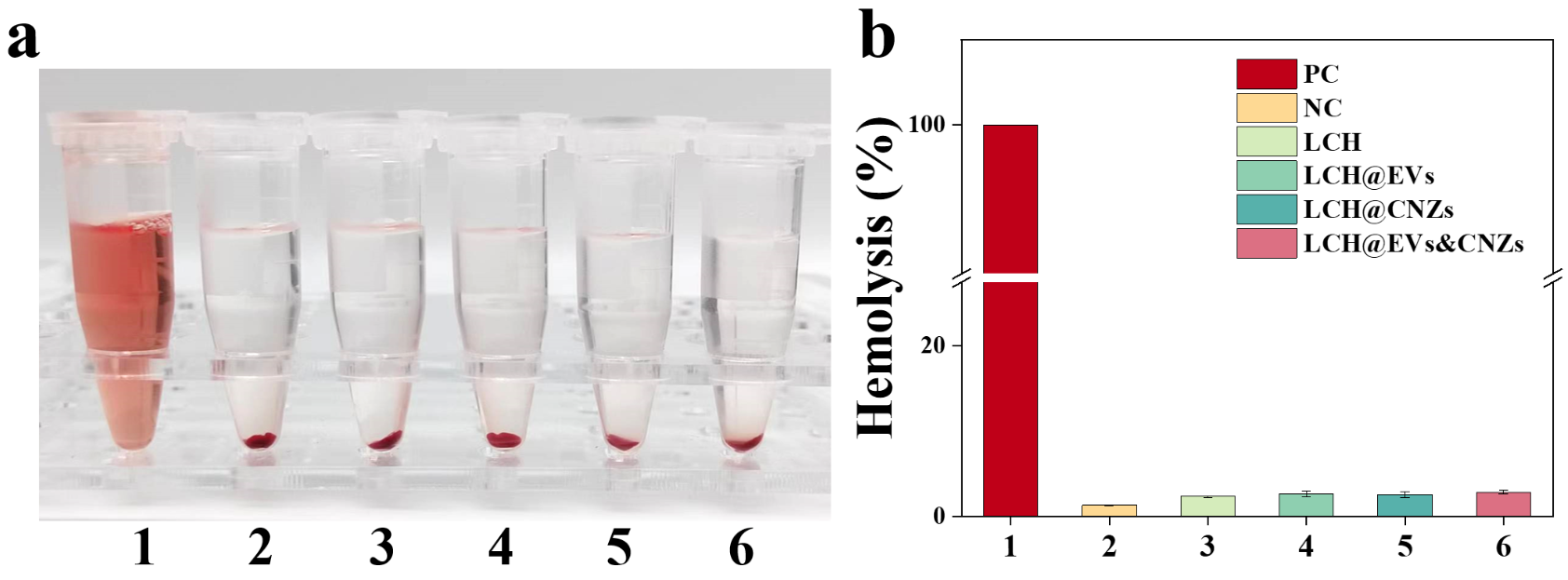


**Fig. S12 Hemolysis test.** 1, Positive control; 2, Negative control; 3, LCH; 4, LCH@EVs; 5, LCH@CNZs; 6, LCH@EVs&NZs.
